# Astrocytes Exhibit a Protective Role in Neuronal Firing Patterns under Chemically Induced Seizures in Neuron–Astrocyte Co-Cultures

**DOI:** 10.3390/ijms222312770

**Published:** 2021-11-25

**Authors:** Annika Ahtiainen, Barbara Genocchi, Jarno M. A. Tanskanen, Michael T. Barros, Jari A. K. Hyttinen, Kerstin Lenk

**Affiliations:** 1 Faculty of Medicine and Health Technology, Tampere University, 33520 Tampere, Finland; jarno.tanskanen@tuni.fi (J.M.A.T.); michael.barros@tuni.fi (M.T.B.); jari.hyttinen@tuni.fi (J.A.K.H.); kerstin.lenk@tugraz.at (K.L.); 2 School of Computer Science and Electronic Engineering, University of Essex, Colchester CO4 3SQ, UK; 3 Institute of Neural Engineering, Graz University of Technology, 8010 Graz, Austria; 4 BioTechMed, 8010 Graz, Austria

**Keywords:** 4-AP, gabazine, epilepsy, astrocytes, MEA, co-cultures

## Abstract

Astrocytes and neurons respond to each other by releasing transmitters, such as γ-aminobutyric acid (GABA) and glutamate, that modulate the synaptic transmission and electrochemical behavior of both cell types. Astrocytes also maintain neuronal homeostasis by clearing neurotransmitters from the extracellular space. These astrocytic actions are altered in diseases involving malfunction of neurons, e.g., in epilepsy, Alzheimer’s disease, and Parkinson’s disease. Convulsant drugs such as 4-aminopyridine (4-AP) and gabazine are commonly used to study epilepsy in vitro. In this study, we aim to assess the modulatory roles of astrocytes during epileptic-like conditions and in compensating drug-elicited hyperactivity. We plated rat cortical neurons and astrocytes with different ratios on microelectrode arrays, induced seizures with 4-AP and gabazine, and recorded the evoked neuronal activity. Our results indicated that astrocytes effectively counteracted the effect of 4-AP during stimulation. Gabazine, instead, induced neuronal hyperactivity and synchronicity in all cultures. Furthermore, our results showed that the response time to the drugs increased with an increasing number of astrocytes in the co-cultures. To the best of our knowledge, our study is the first that shows the critical modulatory role of astrocytes in 4-AP and gabazine-induced discharges and highlights the importance of considering different proportions of cells in the cultures.

## 1. Introduction

Astrocytes are glial cells that control and sustain homeostasis in the central nervous system (CNS). In this study, we aim to investigate the role of astrocytes in the homeostasis of neuronal activity after the application of commonly used chemicals to induce epileptiform behavior. Adjacent astrocytes are connected via gap junctions (GJs) that mainly comprise connexins. GJs maintain brain functions, e.g., by channeling the propagation of adjoining astrocytic calcium (Ca^2+^) waves and regulating ion flow, neuronal activity, and behavior [1]. Astrocytes and neurons are in constant bidirectional feedback-like communication with each other. The interconnected group of a pre- and post-synaptic neuron and the astrocyte is referred to as tripartite synapse [2]. Neuronal firing induces the release of neurotransmitters like glutamate and γ-aminobutyric acid (GABA) from the pre-synaptic neurons [3,4]. When glutamate binds to the metabotropic glutamate receptors (mGluRs) at the astrocytic cell membrane, a cascade of chemical reactions elicits inositol trisphosphate (IP_3_) production, which triggers intracellular [Ca^2+^]_i_ transients [5]. Astrocytes also possess GABA_A_ receptors, which are structurally comparable to those of the neurons [6]. However, the role of GABA_A_ in astrocytes is still unclear; previous research reported that GABA_A_ receptor (GABA_A_R) activation depolarizes the astrocyte’s membrane and activates voltage-gated Ca^2+^ channels and evokes astrocytic [Ca^2+^]_i_ transients [7,8]. The GABA uptake activates GABA:Na^+^ co-transport by GAT-3, resulting in an accumulation of intracellular Na^+^. Astrocyte [Ca^2+^]_i_ transients are elicited by the Na^+^/Ca^2+^ exchanger and the relative Ca^2+^-induced Ca^2+^ release from the endoplasmic reticulum [9].

The rise in [Ca^2+^]_i_, elicited by glutamate or GABA uptake, may result in the release of gliotransmitters that regulate the synaptic transmission and synaptic plasticity of neurons [2]. Released glutamate, together with ATP, D-serine, and adenosine, activates pre- and post-synaptic neuronal receptors, modulating the synaptic transmission and enhancing long- and short-term synaptic plasticity [10]. GABA released by the astrocytes induces slow outward currents (SOCs) in the neighboring neurons. The SOCs are inhibitory currents and are elicited by the activation of GABA_A_Rs [11]. Thus, the complex interactions between neurons and these types of glial cells regulates and maintains homeostasis in the brain, e.g., by modulating GABA and glutamate levels [12,13].

Dysfunctions in astrocytes have been connected to several CNS diseases, including epilepsy, Alzheimer’s disease, and Parkinson’s disease [14,15,16,17]. Epilepsy is a chronic disease characterized by seizures that can sometimes appear as involuntary body movements. Seizures can initiate in various parts of the brain and are characterized by excessive electrical discharges in groups of neurons [18]. Epilepsy is a family of complex heterogeneous disorders with multiple possible underlying causes, such as different channelopathies; or trauma, such as traumatic brain injury [19]. Due to the progression of the disease and the increased severity of the symptoms with time, as well as the increased cognitive and physical impairments in patients, epilepsy is often associated with neurodegenerative disorders [20]. In addition, a recent population-based study confirmed that epilepsy was more often diagnosed in patients affected by Alzheimer’s disease or Parkinson’s disease [21].

The central astrocytic dysfunctions linked to epilepsy concern the impaired extracellular potassium (K^+^) buffering and the excessive neurotransmitters in the extracellular space (ECS) [16]. Increased levels of K^+^ can impair ionic homeostasis in the ECS, affecting the regular ionic exchanges through the membranes. Furthermore, elevated levels of glutamate stimulates excessive IP_3_ production, thus affecting the astrocytic [Ca^2+^]_i_ transients. Moreover, GABA has a central role in epilepsy, and disturbances in the glutamine–glutamate–GABA metabolic pathways may lead to seizures. Such GABA-mediated faults include, for example, the loss of GABAergic interneurons, the overproduction of GABA by reactive astrocytes, and occurring GABA_A_R -mediated tonic inhibition [22].

Epilepsy has been widely studied in vitro by reproducing the specific characteristics of epileptic seizures. These features are commonly investigated using, e.g., brain slices or cultures of primary rodent cells, as well as human induced pluripotent stem cells. Therefore, the neuronal extracellular activity is often recorded with microelectrode array (MEA) measurements [23,24,25]. MEA electrodes record extracellular potentials of neurons or neuronal populations in the vicinity of the electrodes. MEA recordings can be analyzed to study, for example, spiking and bursting activity patterns and action potential waveforms across the whole MEA area or at a subset of electrodes [26].

The brain exhibits an inhomogeneous distribution of neurons and astrocytes throughout the different brain areas [27]. In the human cortex, the relative amount of astrocytes amongst all the glial cells is approximately 50% [28]. In some studies with MEAs, electrophysiological recordings of cultures with only neurons, and co-cultures of neurons and astrocytes, were compared [29,30]. However, the numbers of plated astrocytes and neuronal cells are often fixed, i.e., the studies do not consider the possible effects of the neuron–astrocyte ratio. In previous studies, neuronal cultures supported with astrocytes have shown increased neuronal viability [31], earlier neuronal network formation and maturation [29], and differing firing rates [29,30] compared to ‘pure’ neuronal cultures, where no astrocytes were explicitly added. However, to our knowledge, there have been no studies where the implications of the neuron–astrocyte ratio on neuronal activity have been investigated in a systematic manner.

Two convulsant drugs, 4-aminopyridine (4-AP) and SR-95531 hydrobromide (gabazine), are often used to induce and study epilepsy in in vitro models. However, the role of astrocytes in neuronal dynamics under the influence of these convulsants remains uncertain. 4-AP is a non-selective voltage-gated K^+^ channel blocker that has been reported to cause synchronous neuronal firing, increase action potential duration, and increase neuronal excitability by affecting the repolarization of the neuronal cell membrane after the depolarization phase of an action potential [32,33]. 4-AP induces epileptiform activity in neurons and results in synchronized [Ca^2+^]_i_ transients modulated by GABA and glutamate receptors [34,35,36,37]. 4-AP likely affects the behavior of astrocytes since 4-AP-mediated stimulation induces an [Ca^2+^]_i_ rise, and at high concentrations, a sequestration of [Ca^2+^]_i_ of both excitable and non-excitable cells [35,38].

Gabazine is a competitive GABA_A_R antagonist that is reported to increase spike rates, induce changes in neuronal firing patterns to more synchronized bursts, and cease slow negative potentials [39,40]. It allosterically inhibits the chloride ion (Cl^-^) influx and therefore reduces GABA-mediated synaptic inhibition. Thus, gabazine causes epileptiform activity in neurons by inhibiting neuronal hyperpolarization [41,42]. Therefore, mutations in extrasynaptic GABA_A_R subunits are linked to epilepsy [43].

While researchers have studied the effects of 4-AP and gabazine on neurons, the role of astrocytes in these networks still needs further investigation. Hence, our study aims to explore how the neuronal responses to these drugs vary depending on the astrocyte numbers in the culture.

In this study, we investigated the modulatory effects of astrocytes on neuronal function during pharmacologically induced epileptiform discharges. We thus plated rat-derived neuron–astrocyte co-cultures and compared them with neuron cultures without added astrocytes. To differentiate between ion-elicited and transmitter-elicited epileptic-like activity, we induced hyperactivity in our cultures using 4-AP and gabazine, respectively, at 28 days in vitro (DIV). We hypothesized that a larger share of astrocytes in the culture would influence neuronal network development and the ionic and neurotransmitter homeostases, which would affect the responses to gabazine and 4-AP. To this end, we examined the effects of the astrocytic control on drug-induced epileptiform activity in cultures with different relative numbers of astrocytes and neurons. We quantified the differences in the activities of the baseline and the drug-induced cultures in terms of the spike rate, burst rate, and the number of active channels during the development (from DIV7 to DIV28). We studied the bursting patterns more deeply before, during, and after the epileptiform activity induction. Furthermore, the effects of these two chemicals on neuron and astrocyte viability were tested after 24 h of stimulation with a live/dead assay. In general, we investigated in our study the effects of the general epileptiform inducers 4-AP and gabazine on the modulatory effects of astrocytes on network activity, and the importance and effects of the neuron–astrocyte cell count ratio on drug responses. With this work, we aimed to elucidate the important role of astrocytes in neuronal function and homeostasis in health and disease.

## 2. Results

The following sections present the results obtained by live/dead assays, the cell counting of the different cell cultures, the spike rate (SR) and burst rate (BR) analyses of the baseline activities due to AP and gabazine stimulation, and the analysis of the synchronization based on cross-correlations and the binned spiking activity. Table 1 summarizes the methods and overall results.

### 2.1. Neuron–Astrocyte Culture Ratios Corresponded Well to the Cell Plating Ratios

**Figure 1:**
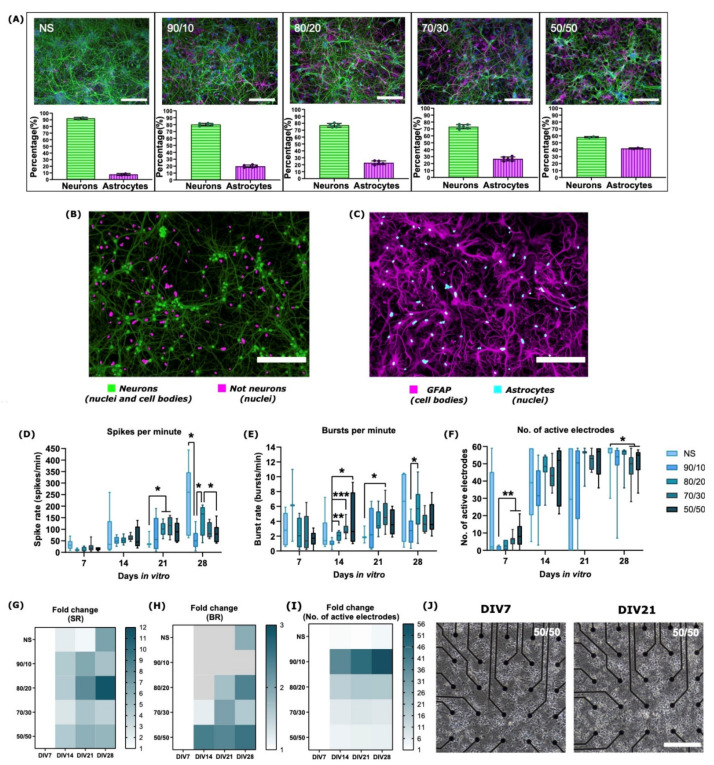
The number of astrocytes in the culture affects (co-)culture structure and neuronal electrical activity and its development. (**A**) ICC images of MAP2-positive neurons (green) and GFAP-positive astrocytes (magenta) of NS and neuron–astrocyte co-cultures, along with the percentages of neurons and astrocytes, which were calculated for each culture. The bar height represents the average percentage of cells, the whiskers the standard deviations, and the dots the individual values for each culture’s region of interest (ROI). Nucleus staining with DAPI is indicated in blue. (**B**) A representative image for counting MAP2-positive neurons and their nuclei (green) and the nuclei of non-neuronal cells (magenta). (**C**) A representative image for counting GFAP- and DAPI-positive astrocyte nuclei (cyan) and the cell bodies of the GFAP-positive astrocytes (magenta). The development of (**D**) neuronal spiking activity, (**E**) bursting activity, and (**F**) number of active electrodes for each culture over 28 days in vitro. In the box plots, the darker blue bar in the middle of each box represents the median, and the attached whiskers range from the smallest value to the largest. * *p* < 0.05; ** *p* < 0.01; *** *p* < 0.001. In general, co-cultures with higher ratios of astrocytes (70/30 and 50/50) had more spiking, bursting, and active electrodes at earlier stages of culture (DIV7-DIV14). However, towards DIV28, the activity of co-cultures stabilized compared to the previous week, but the activity of NS continued to increase. Fold change of (**G**) spike rate, (**H**) burst rate, and (**I**) number of active electrodes after DIV7. Spike rates increased from DIV7 for all the cultures until DIV28, except that the spike rates for 90/10, 70/30, and 50/50 decreased slightly from DIV21 to DIV28. Burst rates developed faster for 80/20, 70/30, and 50/50 from DIV7 to DIV21, whereas NS and 90/10 lagged behind in burst rate development (decreasing fold change depicted in grey). The number of active electrodes exploded from DIV7 to DIV14 for all the cultures but remained relatively steady after that. (**J**) Representative images of DIV7 (left) and DIV21 (right) of the same 50/50 co-culture on MEA. The development of neuronal networks was visually observable with a microscope. Scale bar 200 µm in all images.

We plated neuron cultures without any added astrocytes (NS), as well as neuron–astrocyte co-cultures with cell count ratios of 90/10, 80/20, 70/30, and 50/50. In Figure 1A, representative immunofluorescent images used in cell counting at DIV14 of MAP2-positive neurons and GFAP-positive astrocytes for the NS and co-cultures are shown, along with the percentages of detected neurons and astrocytes. The percentages of neurons were, on average, 92.2, 80.2, 77.1, 73.3, and 58.2% for the NS, 90/10, 80/20, 70/30, and 50/50 cultures, respectively. The standard deviation of MAP2-positive cell counts from the total number of cells was only approximately 4.9% within the cultures, confirming near equal neuronal numbers and densities in all the cultures. The pairwise differences in the neuron counts between cultures were always statistically insignificant (*p* > 0.05), confirming uniform neuronal culture density. The number of astrocytes at DIV14 corresponded to the seeded cell numbers: we detected, on average, 7.8, 19.9, 22.9, 26.7, and 41.8% of astrocytes in the NS, 90/10, 80/20, 70/30, and 50/50 cultures, respectively (Figure 1A). Note that a small percentage of astrocytes already existed in the commercially obtained neuronal cell stock in the NS cultures. Representative images of the masks created by the in-house semi-automated MATLAB tool are shown in Figure 1B,C.

### 2.2. The Development of Neuronal Baseline Activity in NS vs. Neuron–Astrocyte Co-Cultures

Spontaneous baseline electrical activity gradually increased in all cultures in a typical manner (Figure 1D–F) from DIV7 onward until the chemical stimulation was applied at DIV28. At DIV7, all cultures on the MEAs were active, but spiking and bursting activities were low. Co-cultures containing more astrocytes exhibited enhanced early-stage development and maturation of the neuronal networks, evaluated by their spike and burst rates (SRs and BRs), and numbers of active channels. In the earlier stages of the culture (DIV7 and DIV14), the SRs and BRs of the co-cultures developed faster, the numbers of active electrodes were higher, and, overall, the variability of the electrical activity across the MEA channels of the co-cultures were lower than for the NS (Figure 1D–F). Co-cultures with 50/50 and 70/30 ratios had more active electrodes than co-cultures containing less astrocytes. For instance, for the 90/10 ratio at DIV7, there was less than one active electrode on average, whereas the values for the 70/30 and 50/50 ratios were about six (*p* = 0.005; Mann–Whitney U test) and nine (*p* = 0.005), respectively. Between DIV14 and DIV21, neuronal electrical activity increased in all cultures and reached its peak around DIV28 (Figure 1D–F). At DIV14, co-cultures containing a higher astrocyte ratio exhibited more bursting than the 90/10 ratio and NS cultures. In fact, at DIV14, the average burst rate for the 80/20 ratio was almost two times higher, the 70/30 ratio almost three times higher, and the 50/50 ratio almost four times higher compared to the 90/10 ratio (*p* = 0.003, *p* = 0.0006, and *p* = 0.01 for the 80/20, 70/30, and 50/50 ratio co-cultures, respectively). At DIV21, both SRs and BRs were higher for co-cultures compared to the NS cultures. Both the 80/20 and 70/30 ratio co-cultures had twice more spikes on average than the NS cultures (*p* = 0.05 for both), and the 70/30 ratio had over 2.3 times more bursts than the NS (*p* = 0.02). At DIV28, there were slightly more active electrodes for the NS cultures compared to the 70/30 (*p* = 0.04) and 50/50 ratio (*p* = 0.04) co-cultures. Moreover, the NS cultures had more spiking activity on average compared to the 90/10 ratio culture (*p* = 0.03). The 80/20 ratio co-culture had both more spikes (*p* = 0.01) and bursts (*p* = 0.04) per minute on average compared to the 90/10 ratio co-culture. Moreover, the 80/20 ratio co-culture had 144 spikes per minute on average, whereas the 50/50 ratio co-culture had 83 spikes per minute (*p* = 0.05).

The NS and co-cultures experienced maturation at different rates. Heat maps in Figure 1G–I present the fold change of the SR, BR, and the number of active electrodes of each culture at different weeks in vitro compared to DIV7. Representative images of one 50/50 neuron–astrocyte co-culture on the MEA are presented in Figure 1J, showing the stage of the same network at DIV7 and DIV21.

From DIV7 to DIV14, the NS cultures experienced the slowest increase in the spike rate (114% and a 2-fold increase) compared to neuron–astrocyte co-cultures with over 300% and a 4-fold increase in their spike rates, except for the 70/30 ratio co-culture (Figure 1G). Moreover, the number of active electrodes drastically increased from DIV7 to DIV14 and rose from 7 to 41 electrodes on average for all cultures (Figure 1I). At DIV7, the 90/10 ratio co-culture had only one active electrode, whereas at DIV14, there were 33 active electrodes, resulting in higher change compared to other cultures (Figure 1I). However, the average SRs and BRs remained lower for the 90/10 ratio co-culture at DIV14 compared to other co-cultures with more astrocytes (Figure 1D,E). Therefore, the 90/10 ratio co-cultures lagged in maturation despite the high increase in the fold change of the active electrodes. Furthermore, only the 70/30 and 50/50 co-cultures with a higher ratio of astrocytes experienced an increase in BRs (20% and a 1.2-fold increase for the 70/30 ratio co-culture and 146% and 2.5-fold increase for the 50/50 ratio co-culture), whereas BRs slightly decreased for other cultures from DIV7 to DIV14 (Figure 1H, where the decrease is depicted in a grey color). At DIV21, all co-cultures had above a 1-fold change in SRs, BRs, the number of active electrodes compared to DIV7, and a percentage increase from DIV14 values, except for the 50/50 ratio co-culture that had 4% less bursting compared to DIV14, and the 90/10 ratio co-culture that had lower BR compared to DIV7. At DIV28, SRs for all cultures increased compared to DIV7, but percentage-wise there was a decrease from DIV21 for the 90/10, 80/20, and 50/50 ratio co-cultures (−25%, −17%, and −5%, respectively). Therefore, co-cultures with higher ratios of astrocytes, the 70/30 and 50/50 ratio co-cultures in particular, experienced relatively earlier maturation in terms of fold changes in SRs, BRs, and the number of active electrodes, which was evaluated by a more robust increase in their activity at the early stage of culture (from DIV7 to DIV14). However, the activity of co-cultures stabilized towards 28 days in the culture, whereas the NS cultures experienced a continued increase in activity at the later stages of culture (from DIV21 to DIV28) after a relatively slow start.

### 2.3. 4-AP and Gabazine Affected Neuronal Activity but Did Not Decrease Cell Viability

**Figure 2:**
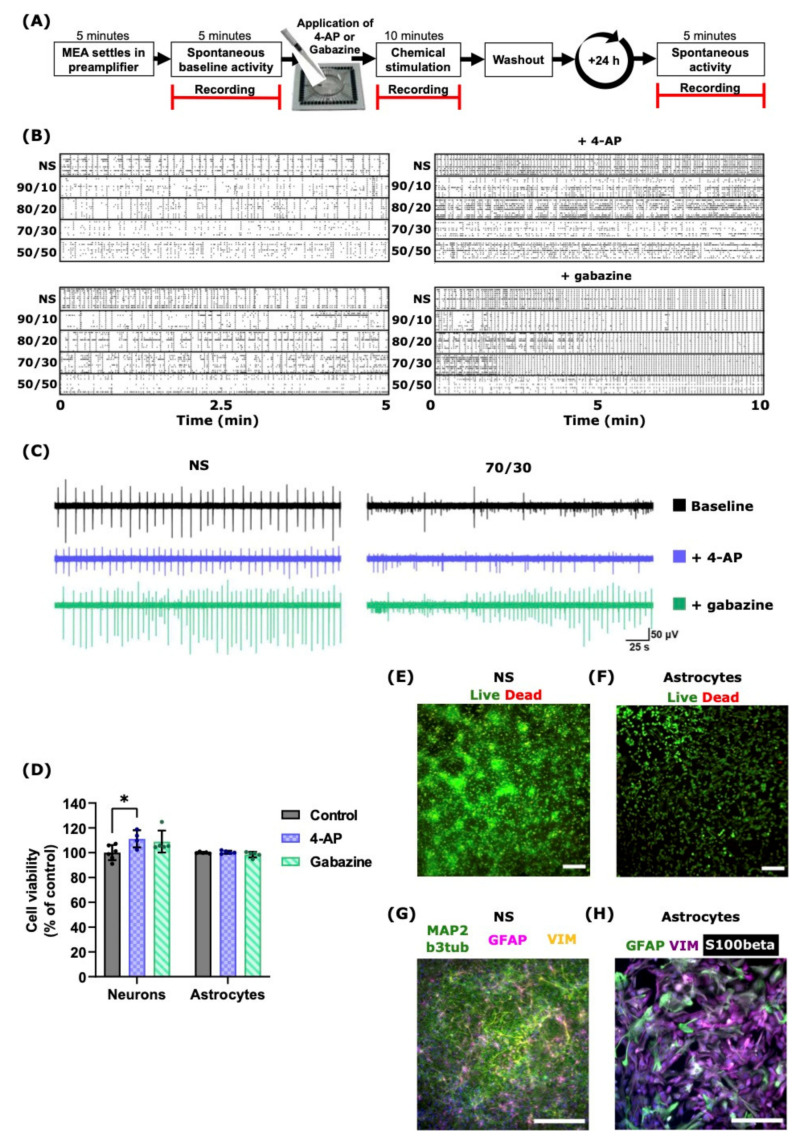
Functional and morphological assessments of primary neuron and astrocyte cultures. (**A**) Measurement protocol for cultures on MEAs: all MEAs were left to settle for 5 min before recording the spontaneous (baseline) activity. Neurons and co-cultures were directly stimulated on MEAs by either 75 µM 4-AP or 30 µM gabazine, followed by a 10-min recording right after stimulation. Convulsants were washed out, and the cell culture medium was replaced. The next day, spontaneous activity and culture recovery were evaluated with a 5-min spontaneous recording. (**B**) Raster plots show different cultures’ baseline activity before (left panels) and after 4-AP or gabazine exposure (right panels). (**C**) Representative neuronal spiking activity after 4-AP (violet) or gabazine (green) stimulation and at baseline (black) for NS (left traces) and 70/30 cultures (right traces). (**D**) Neuron and astrocyte viability assessment results indicated that neither 4-AP nor gabazine caused cell death. In fact, 24-h 4-AP exposure increased the viability of NS cultures (*p* = 0.038). (**E**,**F**) Representative live/dead images of rat cortical neurons and astrocytes. Live cells are shown in green and dead cells in red. (**G**,**H**) Representative, untreated, ICC images of rat cortical neurons and astrocytes. Astrocytes uniformly expressed astrocyte-specific markers GFAP (**G**) violet, (**H**) green), S100β (white), and vimentin (**G**) yellow (**H**) violet) as well as neuronal proteins such as β3-tubulin and MAP2 (**G**) both in green). Nucleus (DAPI) is indicated in blue. Scale bar 200 µm in all images.

The MEA recordings and the chemical stimulation workflow are presented in Figure 2A and are described in the Method Section 4.5 and Section 4.6.

The possible cytotoxic effects and phenotypic impacts of 4-AP and gabazine were tested with a live/dead assay (Supplementary Figure S2A,B) and immunocytochemistry (ICC) (Supplementary Figure S2A,B). The live/dead assay confirmed that neither 4-AP nor gabazine affected astrocyte viability after 24 h from the exposure compared to unstimulated control cultures (Figure 2E,F). Moreover, for the NS cultures, 4-AP increased neuronal viability by approximately 10% (*p* = 0.04; Mann–Whitney U test) compared to unstimulated cultures, whereas gabazine did not cause significant changes (Figure 2D).

The effects of the chemicals were also observed using ICC, which confirmed no visible changes in the neuron (Figure 2G) or astrocyte (Figure 2H) morphology, which was evaluated from the expressions of neuron- and astrocyte-specific markers, i.e., GFAP, S100β, and vimentin (VIM) for astrocytes (Figure 2H), and MAP2 and β3-tubulin (b3tub) for neurons. Hence, neuron and astrocyte cultures had their typical ICC marker expression without any apparent signs of phenotypic alterations after 4-AP or gabazine exposure. Moreover, almost all MEAs still presented spiking and bursting activity after 24 h from the exposure to the drugs (Supplementary Figure S1A–D).

The representative acute effects of 4-AP and gabazine on the neuronal spike activity are shown in Figure 2B. The left panels show the raster plots of the spontaneous activities of the different NS and co-cultures before the chemical stimulation; the activity of the corresponding MEAs after 4-AP or gabazine exposure is shown in the raster plots in the right panels. The convulsant 4-AP increased the SRs and slightly decreased burst synchronization (Figure 2B, upper right), while gabazine induced hyper-synchronized activity in the networks (Figure 2B, lower right). A deeper analysis of burst synchronization is shown in Section 2.4. Figure 2C illustrates the changes in the neuronal activity of the NS and 70/30 ratio co-cultures before and after chemical stimulation: 4-AP did not cause any visible changes in neuronal spiking, whereas gabazine affected both the NS and 70/30 ratio co-cultures by increasing the neuronal activity and spiking intensity.

**Figure 3:**
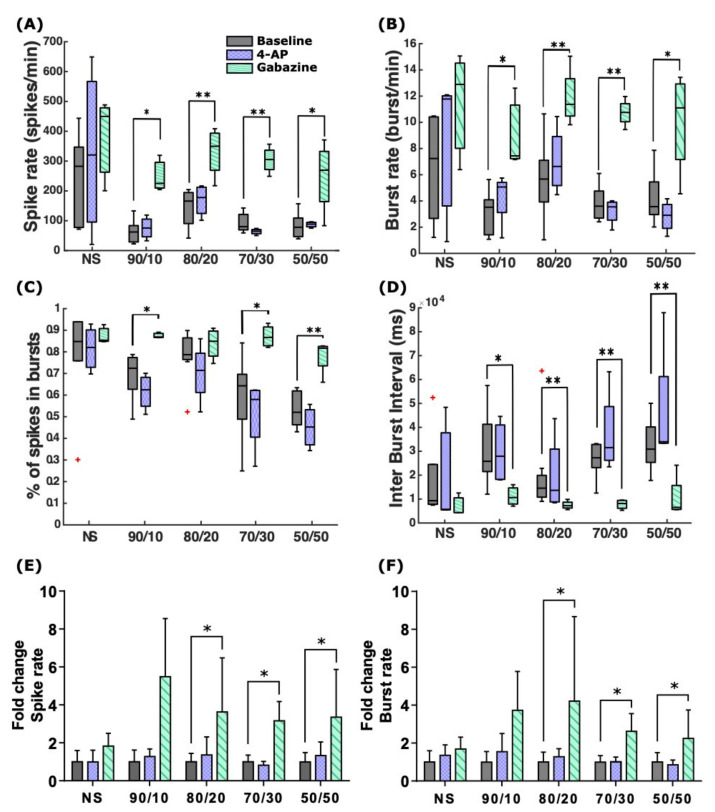
Responses of NS and neuron–astrocyte co-cultures to 75 µM 4-AP and 30 µM gabazine at DIV28. (**A**) SR and (**B**) BR. 4-AP slightly increased the SRs and BRs in NS, 90/10, and 80/20 co-cultures but had hardly any effect in the co-cultures with higher numbers of astrocytes. Gabazine increased the SRs and BRs in all cultures. (**C**) The percentages of spikes contained in bursts decreased after 4-AP application in all cultures. However, gabazine induced an increase in the percentage of spikes in bursts in all the cultures. (**D**) The IBIs remained the same or slightly rose after the 4-AP application. Gabazine induced a decrease in IBI, and the decrease was greater in co-cultures with more astrocytes. (**E**,**F**) The fold change in the SRs and BRs compared to the corresponding baseline values before stimulation. * *p* < 0.05; ** *p* < 0.01. In the box plots, the data points for each subplot are the respective MEA averages between the channels; the black bar in the middle of the box represents the median, and the box edges represent the 25th and 75th percentiles, respectively. The whiskers extend to the most extreme data points that are not considered outliers. Red crosses represent outlier cultures. In the fold changes, the bar height represents the population mean, and the whiskers represent the population’s standard deviation.

All cultures on the MEAs exhibited bursting at DIV28 (Figure 3). In general, before the stimulation, the co-cultures with higher numbers of astrocytes showed lower spike and burst rates (Figure 3A,B). Moreover, the percentage of spikes in bursts decreased with the increasing number of astrocytes in the cultures, whereas inter-burst intervals (IBIs), i.e., the time between the end of the previous burst and the start of the next one, increased (Figure 3C,D).

Interestingly, 4-AP seemed to have a varying effect on the cultures depending on the neuron–astrocyte ratios. Cultures with higher numbers of astrocytes exhibited minor effects of 4-AP: 4-AP slightly increased or left almost unchanged the SRs and BRs for the NS, 90/10 ratio, and 80/20 ratio co-cultures. The SRs decreased for the 70/30 ratio co-culture and slightly increased for the 50/50 ratio co-culture, and the BRs decreased for the 50/50 ratio co-culture and remained almost unchanged for the 70/30 ratio co-culture (Figure 3A,B,E,F). However, none of the co-cultures with astrocytes reached a SR comparable with seizure-like hyperactivity. Moreover, the stimulation with 4-AP reduced the percentage of spikes in bursts in all the cultures and slightly increased the IBI and its variability in the co-cultures (Figure 3C,D). These differences, however, were not statistically significant.

Gabazine stimulation had a much greater effect on neuronal activity than 4-AP stimulation. Gabazine increased the SRs and BRs in all the cultures (Figure 3A,B; *p* = 0.02 SRs and BRs for the 90/10 ratio co-culture; *p* = 0.004 SRs/0.008 BRs for the 80/20 ratio co-culture; *p* = 0.004 SRs and BRs for the 70/30 ratio co-culture; *p* = 0.03 SRs/0.02 BRs for the 50/50 ratio co-culture; Mann–Whitney U test). The SR and BR fold change for the NS was lower than for the co-cultures (Figure 3E,F). Gabazine also decreased the IBI values for all the cultures (with an overall average fold change of 0.4; *p* = 0.03 for the 90/10 ratio co-culture; *p* = 0.008 for the 80/20 ratio co-culture; *p* = 0.004 for the 70/30 ratio co-culture; and *p* = 0.008 for the 50/50 ratio co-culture) and increased the percentage of spikes in bursts (with an overall average fold change of 1.3), synchronizing the network activity (Figure 3C,D). Moreover, the relative changes in the percentage of spikes in bursts and IBIs were more notable in co-cultures containing more astrocytes (the 70/30 and 50/50 ratio co-cultures). In all co-cultures, gabazine caused an over 2-fold increase in spike and burst rates (*p* = 0.03, except for the 90/10 and NS cultures), whereas in the NS, the fold change remained under two (Figure 3E,F).

### 2.4. 4-AP and Gabazine Had Different Effects on Neuronal Network Synchronization

**Figure 4:**
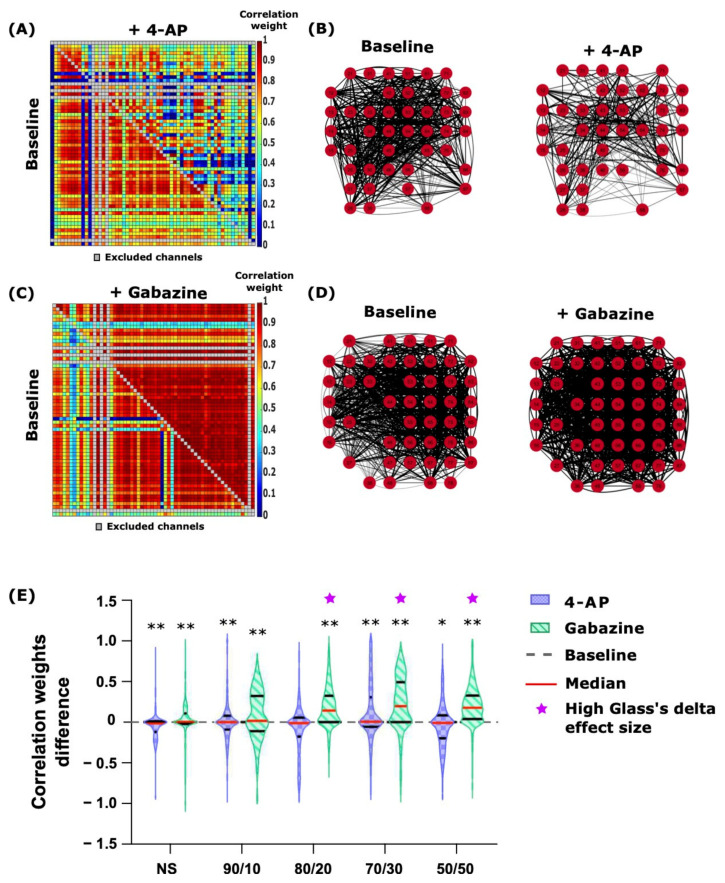
The effects of 75 µM 4-AP and 30 µM gabazine on network connectivity and activity. (**A**,**C**) show the pairwise channel correlation weight heatmaps at baseline (lower-left halves) and after the stimulation (upper-right halves) with 4-AP (**A**) and gabazine (**C**) from a representative 80/20 co-culture on one MEA for each chemical stimulation protocol. Correlation weights span between 0 (blue) and 1 (red). The noisy channel pairs excluded from the analysis and the diagonal points not calculated are shown in grey. (**B**,**D**) show the pairwise channel connectivity maps for the same MEAs and chemical stimulations as in (**A**,**C**), respectively. The red circles represent the MEA channels, and the black lines represent the connections found between the channels. Only the connections with weights larger than 0.75 are shown for clarity. The line thickness represents the correlation weight. (**E**) displays the correlation weight differences after stimulation with 4-AP or gabazine compared to baseline for each channel pair. 4-AP slightly decreased channel correlation weights or left them unchanged, whereas gabazine increased them in all the cultures except NS. The effect was more pronounced for higher numbers of astrocytes. The exact numerical values of the Glass’s delta effect size analysis can be seen in Supplementary Table S1. The grey dashed line represents the weight difference if there are no changes between baseline and chemical stimulation; the solid red lines are the median, the solid black lines are the quartiles, and the magenta stars represent the populations with higher Glass’s delta effect sizes. * *p* < 0.05, ** *p* < 0.01.

The application of 4-AP and gabazine had different effects at the network level. We analyzed the correlations of time-varying spectral entropies between MEA channels (CorSE analysis, Section 4.7.3). 4-AP caused a slight decrease in the number of channels with synchronized activity and in the inter-channel correlation weights of the connections (Figure 4A,B). The inter-channel correlation weights represent the level of synchronicity, expressed as a value from 0 to 1, between a selected pair of channels in the MEA. Figure 4A shows a heatmap of the correlation weights between the 60 channels of the MEA; in the heatmap, the correlation weights at baseline are shown in the lower left half, and in the upper right half, the weights for the same channel pairs after the stimulation with 4-AP in a representative 80/20 ratio culture MEA are represented. It appears clear that certain connections were maintained while others were weakened. This is reflected in the channel connectivity map (Figure 4B), where only the channels with correlation weights higher than 0.75 are shown for clarity (for the same MEA as in Figure 4A). The black lines represent the links between the synchronized channels; the thickness of a line reflects the correlation weight of the connection. The stimulation of 80/20 ratio co-cultures with gabazine shows a clear increase in all correlation weights (Figure 4C, Supplementary Table S1). Thus, there is an overall average of a 1.55-fold increase in the number of channels linked with a correlation weight higher than 0.75 (Figure 4D). However, the changes for both 4-AP and gabazine were not statistically significant culture-wise, even though they are visually noticeable.

The violin plots in Figure 4E show that in the case of 4-AP stimulation, the median of the connection weight differences between the baseline measurements and measurements after stimulation, as well as the whole weight difference distribution, were centered around zero, where the stimulation showed almost no effect on the connectivity. However, the gabazine stimulation increased the connection weights in all the co-cultures with astrocytes and shifted the weight difference distribution towards positive values. Here, we analyzed all the connections weights (approximately 3000) between the channels of each MEA (four per culture group), and, due to the large n of the connection weights (n was approximately 10,000) in each culture group, the Student’s *t*-test gave a *p*-Value well below *p* < 0.01 for the pairwise analysis of the weights after 4-AP and at baseline (*p* < 0.0001 for all cultures, except where *p* = 0.1 for the 80/20 ratio co-culture and *p* < 0.05 for the 50/50 ratio co-culture). The effect size analysis showed a low to medium effect (Glass’s delta ≤ 0.6) of the stimulation with 4-AP. In contrast, in the case of gabazine stimulation, the effect size analysis showed a low effect for the NS (Glass’s delta = 0.15), a medium effect for the 90/10 ratio co-cultures (Glass’s delta = 0.7), and a high effect for the co-cultures with more astrocytes (Glass’s delta ≥ 0.9) (*p* < 0.0001 for all cultures). All the Glass’s deltas resulted in positive values, depicting an increase in the stimulated weights, except in the stimulation with 4-AP in the 80/20 ratio cultures. The Glass’s delta and its confidence intervals for all the comparisons between the stimulated effects and the baseline are shown in Supplementary Table S1.

The network effects of 4-AP and gabazine were also observable in the numbers of network-wide bursts (NBs). Gabazine synchronized the bursting of all the networks in all the cultures; this effect was more pronounced in neuron–astrocyte co-cultures containing higher numbers of astrocytes (Supplementary Figure S1F; *p* = 0.03 for both the 80/20 and 70/30 ratio co-cultures). The increasing number of NBs for gabazine stimulation was directly related to the neuron–astrocyte ratio (1.1, 2.4, 3.8, 12.1, and 7.8-fold changes for the NS, 90/10, 80/20 70/30, and 50/50 ratio co-cultures, respectively). There was no change in the number of NBs in 4-AP stimulation of the cultures (Supplementary Figure S1E). However, 24 h after stimulation, cultures exposed to drugs showed fewer NBs than the day before, except for the NS (*p* = 0.03; Mann–Whitney U test).

### 2.5. Gabazine Induced Synchronization and Neuron–Astrocyte Ratio Effects on Gabazine’s Delay in Response Time

**Figure 5:**
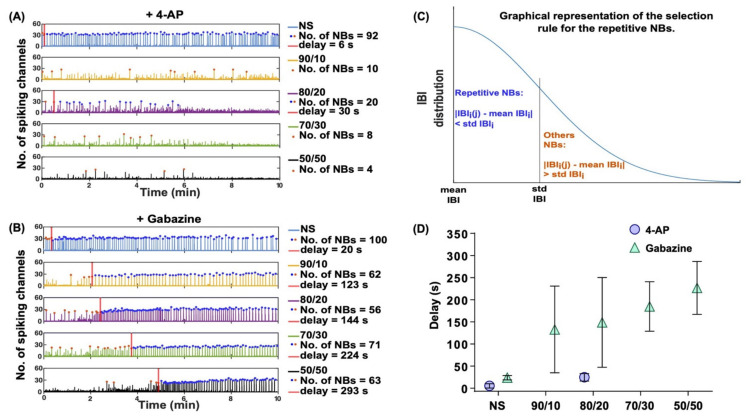
(**A**,**B**) Channel spiking activity summed in 5 ms time bins for five cultures (NS in blue, 90/10 in yellow, 80/20 in violet, 70/30 in green, and 50/50 in black) and the detected NBs (orange dots and blue dots) over the 10-min recordings after stimulation with 75 µM 4-AP and 30 µM gabazine, respectively. The blue dots represent the repetitive NBs, and the orange dots represent the other NBs. When both are presented, the number of NBs is defined as the sum between the two. The *y*-axis of each subplot shows the number of channels participating in an NB. The red lines represent the delays defined as the time points of the first bursts belonging to the repetitive burst series (blue dots). (**C**) shows a graphical representation of the rule we used to select the bursts belonging to the repetitive burst series (blue dots); (i) is the MEA index, and (j) is the NB index in the series. (**D**) shows the delays due to the gabazine (in green) and 4-AP (in lilac) effects on the network activities for each cell culture type.

Interestingly, the effect of gabazine on neuronal activity was delayed in the co-cultures with higher numbers of astrocytes. The NBs in the MEAs stimulated with 4-AP spanned fewer channels (around 20 channels on average) than in the gabazine stimulated NBs (approximately 30 channels on average) (Figure 5A). Moreover, the bursting activity of the stimulated MEAs was not repetitive and did not show any noticeable differences between cultures. The repetitive synchronous NBs in MEAs stimulated with gabazine spanned a constant number of channels, which slightly decreased with the increasing number of astrocytes (Figure 5B).

Lowering the neuron–astrocyte ratio (i.e., increasing the relative numbers of astrocytes) drastically delayed the effects of gabazine on neuronal firing. For the NS, the delay was, on average, around 25 s, whereas it was approximately 130 s for the 90/10 ratio co-cultures, 150 s for the 80/20 ratio co-cultures, 180 s for the 70/30 ratio co-cultures, and approximately 230 s for the 50/50 ratio co-cultures (Figure 5D). Figure 5D shows the delays in all the MEAs for the NS and co-cultures and indicates an increasing delay of gabazine effects with the increasing numbers of astrocytes. The 90/10 and 80/20 ratio co-cultures exhibited a higher inter-culture variability. No delayed effects or changes in the NB synchronization were noticeable for 4-AP (Figure 5A,D).

## 3. Discussion

In our study, we aimed to investigate the role of astrocytes in balancing drug-elicited epileptic activity. We investigated the effects of the numbers of astrocytes, with respect to the number of neurons, on neuronal firing, bursting, and network behavior with and without 4-AP and gabazine application. We anticipated that astrocytic control, determined by the number of astrocytes in the cultures, might enhance early neuronal network development compared to cultures with less astrocytes.

Our study showed that the addition of astrocytes to the neuronal network led to the earlier development and maturation of the cultures based on their electrical activity characterizations, confirming previous findings on primary co-cultures [29]. It has been shown that astrocytes are crucial for the synaptogenesis of neurons, and that they also contribute to neuronal branching and the formation of functional networks with circuits [44]. Therefore, the astrocytic regulation of nascent synapse formation, neuronal network development, and maturation, especially in the early stages of culture (DIV7 to DIV14), are likely to enhance the maturation of the co-cultures containing more astrocytes. Moreover, astrocytes have a significant role in regulating synapse maintenance and elimination that defines the structure of the network. This might explain the lower activity variance across the cultures and the stabilization in neuronal activity of the higher astrocyte-ratio co-cultures towards DIV28 as the neurons and astrocytes begin to show mature morphology.

4-AP and gabazine are two convulsant drugs commonly used in in vitro experiments to induce epileptic seizures in brain tissues [32,33,45]. 4-AP is a voltage-gated K^+^ channel blocker, and gabazine is a GABA_A_ receptor antagonist. 4-AP application results in synchronized [Ca^2+^]_i_ transients, which trigger the release of glutamate [35]. Both neurons and astrocytes express voltage-gated K^+^ channels and GABA_A_ receptors. Therefore, these drugs are expected to affect both cell types.

Gabazine has been shown to block GABAergic signaling in both rat and hPSC-derived networks, leading to increased spike rates, burst rates, and synchronization [39,46,47]. In our study, gabazine induced seizure-like hyperactivity in all our cultures, increasing SRs and BRs, which indicates that our cultures had functional inhibitory systems.

Interestingly, for gabazine stimulation, we noticed that the hypersynchronous bursts appeared delayed for the co-cultures, compared to the neuronal cultures, without added astrocytes. This suggests that the maturational stage, which depends on the astrocyte number in the culture, may also affect the modulational effects of the drug. Furthermore, co-cultures with higher numbers of astrocytes have more neuron–astrocyte interconnections with GJs, that allows for a quick exchange of ions, metabolites, and neuroactive substances that are released by neurons and astrocytes during pharmacological stimulation. More astrocytes in the culture also means more GABA_A_Rs, which likely causes differential responses between the cultures. Astrocytes exhibit a high density of GABA_A_Rs, which are functionally and structurally similar to the neuronal ones [6]. In a study conducted on subventricular zone astrocytes, GABA_A_R activation induced a Ca^2+^ increase through the activation of voltage-gated Ca^2+^ channels [48]. Thus, the blockade of the GABA_A_Rs reduces [Ca^2+^]_i_ transients [8]. Since the inhibitory gliotransmitters release, e.g., D-serine from the astrocytes, which depends on Ca^2+^ elevation [49], we hypothesize that reduced intracellular Ca^2+^ levels lead to a failure in the control of neuronal activity and increased SRs and BRs. Corroborating our hypothesis, a reduction in neuronal [Ca^2+^]_i_ transients, and a relative abnormal neurotransmission, was also noticed in acute models of epileptiform activity in vitro on *Sip1* deficient mice [50], and in ischemia-like conditions in hippocampal neurons and astrocytes during oxygen-glucose deprivation/reoxygenation [51]. We noticed that increasing the number of astrocytes, and therefore most probably the amount of GABA_A_Rs, increased the time needed for the drug to block most of these receptors. Hence, gabazine-elicited synchronization was delayed in the co-cultures with higher percentages of astrocytes in the culture, but it led to epileptic-like hyperactivity in all cultures despite the astrocyte numbers.

In our experiments, 4-AP, instead, failed to show any statistically significant increase in SRs or BRs in our cultures. However, our findings indicate that low concentrations (75 µM) of 4-AP increases neuronal viability, which supports the previous findings about the neuroprotective effects of 4-AP on different neuronal types [52,53,54,55,56]. Moreover, 4-AP seems to prevent glutamate-induced cell death [56]. We also demonstrated that the co-cultures with higher percentages of astrocytes more effectively counteracted the effect of 4-AP on neuronal activity. This might result from a more extended astrocytic network which includes an elevated number of GJs that contribute to the clearing of accumulating K^+^ and [Ca^2+^]_i_. Furthermore, our results for the 4-AP stimulation are in line with a previous study by Tukker et al. [57]. They showed that rat cortical cultures containing 45% astrocytes and human induced pluripotent stem cell derived co-cultures with 10–15% astrocytes remained relatively unaffected by 100 µM 4-AP exposure. They also showed that the 4-AP-induced possible excitability is dependent on the cell source and the proportions of different cell types in the culture. However, the drug-induced effects vary between the models and depend on the cell seeding density, neuronal maturation (DIV), and the different ratios of inhibitory and excitatory cell types in the cultures [45,57]. In addition, a study by Bradley et al. tested multiple pharmacological agents with rat primary cortical neurons (without added astrocytes) [47]. They demonstrated a rise in neuronal activity in 4-AP-treated NS cultures. Both 100 µM 4-AP and 10 µM gabazine treatments caused a decrease in the IBI and an increase in the percentage of spikes in bursts. Although, in our study, 4-AP did not increase the percentage of spikes in bursts. Moreover, one of our studies on a computational neuron–astrocyte network model showed that in heightened hyperactivity, the network with more GJs controlled and downregulated the neuronal activity [58]. A previous study [59] conducted a graph analysis on neuronal cultures with no added astrocytes. The application of 4-AP induced hypersynchronization and increased functional connections. This led to decreased path lengths and increased clustering coefficients.

Astrocytes have been shown to clear K^+^ from the ECS with non-voltage-gated K^+^ channels, e.g., Kir channels, Na^+^/K^+^-ATPase, and Na^+^/K^+^/2Cl^−^ cotransporters [60]. Furthermore, they uptake the excessive glutamate through glutamate transporters [61]. The cleared K^+^ and glutamate are successfully buffered to distal areas through GJs [3,15,62]. The effect of the blockade of voltage-gated K^+^ channels by 4-AP is assumingly counteracted by the astrocytic K^+^ and glutamate buffering, which leads to statistically insignificant changes in our recorded activity features, such as SRs, BRs, IBIs, and burst durations. Here, we hypothesize that while the extracellular K^+^ and glutamate were increasing due to the effect of 4-AP on the neurons, the astrocytes may have kept clearing the excess K^+^ and glutamate via non-voltage-gated K^+^ channels and glutamate transporters. The protection was more visible in co-cultures with higher numbers of astrocytes, thus presenting a more significant amount of non-voltage-gated K^+^ channels and transporters.

The use of defined neuron–astrocyte co-cultures provides us with a method to assess the role of astrocytes in neuronal networks since combining neurons and varying numbers of astrocytes results in different network structures. In fact, our approach could be used to assess the effects of drugs in various brain regions, as they have distinct amounts of astrocytes [28]; this would provide additional information with more anatomical relevance about brain functions. Moreover, co-cultures, such as those used here, with predetermined neuron–astrocyte ratios and the relative results on the drug-elicited epileptic-like hyperactivity, can be used to study different brain areas’ susceptibility to epilepsy. From epileptogenic studies, the hippocampus and the cortex, compared to other brain areas, presented a higher epileptogenic index [63], a parameter that describes the propensity of a brain area to generate rapid discharges. In temporal lobe epilepsy, the most frequently epileptogenic areas are the hippocampus, the amygdala, and the pyriform cortex [64]. A study conducted on African giant rats highlighted how the cortex presents a lower GFAP expression compared to other brain regions such as the cerebellum or the dentate gyrus [65]. Altogether, these studies suggest that brain regions with a lower presence of astrocytes might be more susceptible to seizures. Our results, showing the higher sensitivity of co-cultures with less astrocytes to epilepsy-inducing drugs, are in line with the aforementioned ex vivo and in vivo studies.

Therefore, our study further elucidates the important role of astrocytes in controlling neuronal activity and homeostasis and it provides new insights into the mechanisms of the action of 4-AP and gabazine on neurons and astrocytes. Our results also highlight the importance of considering different proportions of cells, or at least assessing the share of astrocytes in neuronal cultures in drug testing.

## 4. Materials and Methods

The cell cultures and performed experiments, described in detail below, are summarized in Table 1, along with the main results.

### 4.1. MEA and Coverslip Preparation

The day before cell plating, all sterilized MEAs (n = 40, 8 MEAs per each plating ratio) and 13 mm diameter glass coverslips were coated with Poly-D-Lysine (PDL, 0.1 mg/mL, Sigma-Aldrich, St. Louis, MO, USA) for one hour. MEAs and coverslips were washed three times with ultrapure water, dried, and incubated with laminin (L2020, 20 µg/mL, Sigma-Aldrich, St. Louis, MO, USA) overnight at +4 °C. The following day, laminin was aspirated just before the plating cells. MEAs used in this study (models: 60MEA200/30iR-Ti and -ITO and 60ThinMEA200/30iR-ITO, Multichannel Systems MCS GmbH, Reutlingen, Germany) had 60 microelectrodes with 30 µm diameters and 200 µm interelectrode distances in eight by eight layouts.

### 4.2. Cell Culture

Rat primary cortical astrocytes (N7745100, Thermo Fisher Scientific, Waltham, MA, USA) were thawed and plated to a cell culture six-well plate (Nunclon; Sigma-Aldrich, St. Louis, MO, USA) at a density of 20 × 10^4^ cells/cm^2^. Astrocytes were cultured for four days (until confluency) in DMEM/F-12 (with HEPES, L-Glutamine) supplemented with a 1% N-2 supplement, 1% Penicillin-Streptomycin (P/S), and 10% fetal bovine serum (all purchased from Thermo Fisher Scientific, Waltham, MA, USA). To prevent further astrocyte DNA replication and proliferation, confluent astrocytes were treated with Cytosine b-D-arabinofuranoside (ara-c, C1768, 2.5 µM, Sigma-Aldrich, St. Louis, MO, USA) for five days, after which they were considered ready for co-culture plating. Cortical astrocytes were also separately plated on coverslips for immunocytochemical analysis (Section 4.3) and on a 24-well plate for live/dead assay after chemical stimulation (Section 4.4) using the same plating density.

Primary rat cortex neurons (A1084001, Thermo Fisher Scientific, Waltham, MA, USA) derived from E18 rat brains were rapidly thawed. Neurons and Ara-C treated astrocytes were separately centrifuged at 250× *g* for 5 min, resuspended in their own media, and counted. The cell count and viability were determined with the trypan blue exclusion assay using the Countess Automated Cell Counter (Thermo Fisher Scientific, Waltham, MA, USA). To achieve different neuron–astrocyte co-culture ratios, the number of neurons was kept constant (80,000 neurons per MEA, 40.000 per coverslip), and the number of astrocytes was adjusted accordingly. The used neuron–astrocyte co-culture ratios were 90/10, 80/20, 70/30, and 50/50 percent (the first number indicates the percentage of neurons, and the second number indicates the percentage of astrocytes). The mixed co-cultures were then recentrifuged before plating. In addition, cultures were prepared with only neurons without separately added astrocytes. These cultures with no separately added astrocytes are referred to as the NS cultures. All used cell cultures for MEAs and coverslips were obtained from a single thaw of cells, i.e., the neuron and astrocyte stocks were from single lots. Neurons and astrocytes were thawed later for 24-h drug treatment characterization (L/D assay and ICC), but these cells had identical lot numbers to the neurons and astrocytes previously plated on MEAs and coverslips.

The NS were plated on coverslips for live/dead assays. In addition, the NS and the co-cultures were plated on the laminin-PDL-coated MEAs and coverslips (for immunocytochemistry (ICC)) in a small drop of medium, which was replenished after cell attachment with either the neuronal medium (NS) or the co-culture medium (co-cultures). The co-culture medium contained neurobasal plus medium with 2% B-12 Plus supplement, 1% P/S, 1% GlutaMAX supplement, and 1% sodium pyruvate. For NS, the same cell culture medium was used without sodium pyruvate and with a 0.25% GlutaMAX supplement. The cell culture medium and all the supplements were purchased from Thermo Fisher Scientific (Waltham, MA, USA). Half the volume of the medium was refreshed every 2–3 days and always after MEA recordings and chemical stimulation. All cell cultures were cultured inside a +37 °C incubator in a 5% CO_2_ atmosphere.

### 4.3. Immunofluorescence and Imaging

Three to four coverslips for each of the co-cultures and the NS were fixed for ICC weekly during the whole culture period and after chemical stimulation (Section 4.4 and Section 4.5). Cells were rinsed with phosphate-buffered saline (D-PBS, Thermo Fisher Scientific, Waltham, MA, USA), and were fixed with 3.7% paraformaldehyde in PBS for 20 min at room temperature (RT). Coverslips were then rinsed with PBS and permeabilized with 0.3% Triton X-100 (Sigma-Aldrich, St. Louis, MO, USA) in PBS for 10 min at RT. After permeabilization, cells were blocked with 5% (*v*/*v*) goat serum (GS, Sigma–Aldrich, St. Louis, MO, USA) in PBS for 120 min at RT and incubated with primary antibodies diluted to 5% (*v*/*v*) GS-PBS overnight at +4 ºC. The primary antibodies used were Microtubule Associated Protein 2 (MAP2, mouse, MAB3418, 1:1000) and Glial Fibrillary Acidic Protein (GFAP, rabbit, AB5804, 1:1000) from Sigma Aldrich (St. Louis, MO, USA). Moreover, GFAP (mouse; MA5-12023, 1:200), S100β (rabbit, PA5-78161, 1:500), β3-tubulin (β3tub, mouse, MA1-118, 1:1000), and Vimentin (VIM, chicken, PA1-10003, 1:1000) were used (Thermo Fisher Scientific, Waltham, MA, USA). The next day, coverslips were washed three times with a wash buffer (0.1% Triton X-100 in PBS) and incubated with species-specific Alexa Fluor conjugated secondary antibodies in the dark for one hour at RT. Secondary antibodies were Alexa fluor 488 (anti-mouse, A-11001, 1:500), Alexa fluor 555 (anti-rabbit, A-21428, 1:500), and Alexa fluor 647 (anti-chicken, A32933, 1:500) (all from Thermo Fisher Scientific, Waltham, MA, USA). Coverslips were washed with the wash buffer, after which 1:1000 4′,6-diamidino-2-phenylindole (DAPI, 10 µg/mL in PBS, Thermo Fisher Scientific, Waltham, MA, USA) was added. After 10 min at RT, the samples were rinsed with PBS, after which coverslips were mounted with a ProLong Gold Antifade Mountant (Thermo Fisher Scientific, Waltham, MA, USA) onto microscope glasses and cured for 24 h in the dark at RT. Coverslips were stored at +4 ºC and imaged with an Olympus IX51 Fluorescence Microscope with an Olympus DP30BW camera (Olympus Corporation, Hamburg, Germany). Images were processed using Fiji (ImageJ) software and analyzed with an in-house MATLAB (MathWorks, Inc., Natick, MA, USA) tool (Section 4.7).

### 4.4. Neuron and Astrocyte Viability

To determine the effect of 4-AP and gabazine on neuron (DIV14) and astrocyte (DIV3) viability, a live/dead Viability/Cytotoxicity Kit (L3224; Thermo Fisher Scientific, Waltham, MA, USA) containing 4 µM ethidium homodimer-1 (EthD-1) and 2 µM calcein-AM was used according to the manufacturer’s instructions. Neurons and astrocytes previously separately plated on 24-well plates and coverslips were chemically stimulated (Section 4.5.) for approximately 24 h. Live/dead imaging of the control and chemically treated cells were captured with a Nikon Eclipse Ti2 (Nikon Instruments, Inc., Melville, NY, USA) fluorescent microscope with an ORCA-Fusion camera (model: C14440-20UP, Hamamatsu Photonics K. K., Hamamatsu City, Japan). The live/dead images were analyzed for cell viability using the particle size-based analysis (modified from [66]). An average of four to six ROIs in total were counted from two to four wells per NS/astrocytes in Fiji software (ImageJ). After chemical stimulation and live/dead assays, neurons and astrocytes on coverslips were fixed for ICC (Section 4.3).

### 4.5. Chemical Stimulation

At DIV28, the co-cultures and the NS on MEAs were stimulated with 75 µM 4-Aminopyridine (4-AP; 0940; Tocris, Bristol, UK) or 30 µM SR-95531 hydrobromide (gabazine; 1262; Tocris, Bristol, UK). Before chemical stimulation, the cells on the MEAs were left to settle in the MEA2100-System preamplifier (Section 4.6) for five minutes to alleviate possible effects of mechanical disturbance from moving the MEAs from the incubator to the preamplifier. After that, spontaneous neuronal activity was recorded for five minutes. Subsequently, appropriate volumes of previously prepared stock solutions (4-AP: 10 mM; gabazine: 25 mM; both diluted in ultra-pure water) of the drugs were pipetted directly to the MEA wells to result in the final drug concentrations, and the cell cultures on the MEAs were immediately recorded for ten minutes. Finally, the whole medium reservoir was replaced with a fresh prewarmed cell medium. The same chemical stimulation replacement was applied on the 24-well plates and coverslips that were stimulated for approximately 24 h inside an incubator (Section 4.4).

### 4.6. MEA Recordings

The electrical activity of the co-cultures and the NS on the MEAs were recorded weekly using a MEA2100-System and the Multichannel Experimenter software (Multichannel Systems MCS GmbH, Reutlingen, Germany). The cultures on the MEAs were left to settle for five minutes in the MEA preamplifier, after which spontaneous neuronal activity was recorded for five minutes. At DIV28, the co-cultures and NS were also chemically stimulated with 75 µM 4-AP or 30 µM gabazine, as described in Section 4.5. Right after chemical stimulation, electrical activity was recorded for ten minutes. After the recordings, the whole medium reservoir was replaced with a fresh cell culture medium, and MEAs were put back into the incubator. After 24 h, MEAs were recorded for 5 min after the 5-min settling period. Raw signals were recorded at the sampling rate of 25 kHz and stored on a personal computer. The protocol to study the induction of epileptiform activity in the cultures is presented in Figure 2A.

### 4.7. Data Analysis

#### 4.7.1. Cell Count

To verify that the relative amounts of neurons and astrocytes in all cultures remained after plating, we counted the cells on the coverslips that we fixed and immunostained with DAPI, MAP2, and GFAP at DIV14 (see Section 4.3). Several ROIs from a coverslip were selected for imaging spanning between the center of the culture and the less populated borders. To count the cells, we developed a semi-automated tool in MATLAB (MathWorks, Inc., Natick, MA, USA). The tool creates segmented black/white (BW) masks of the DAPI-stained nuclei of both cell types. Then, it selects the intensity peaks in the image and removes as debris all continuous regions with radiuses smaller than a predefined radius around the intensity peak. This method also separates the cells grouped in clusters. Thereafter, the revised nuclei are counted.

Then, the mask indicating the nuclei was merged with the mask created from the corresponding MAP2 image, indicating the neurons and their nuclei; if more than a predefined fraction of the nucleus border was contained within the MAP2 mask, the nucleus was considered to belong to a neuron. The threshold was chosen based on the image brightness and visual control to maximize the neuronal detection and minimize the false classification. All the nuclei that were not counted as neuronal were then merged with the GFAP-stained BW mask of the astrocytes. If the still non-categorized nuclei borders were contained in the GFAP mask, whose threshold selection was similar to the one for the MAP2 mask, the cells were counted as astrocytes.

#### 4.7.2. Spiking and Bursting Analysis

The raw MEA signals were filtered, neuronal action potentials detected, and spike waveform cut-outs were sorted with a widely used tool, Wave Clus for MATLAB [67]. The signal was filtered with a second-order bandpass (300–3000 Hz) elliptic filter to retain only the action potential peaks for further analysis. Positive and negative spikes were detected with a threshold of ±5σ, where *σ = median (*$\left|x\right|/0.6745$*)* and *x* is the bandpass filtered signal. The detector dead time was 1.5 ms after each spike.

The timestamps of the spikes obtained from the sorted signals were then further analyzed with a MATLAB tool developed in our group [68]. The tool uses a cumulative moving average-based algorithm [69] further adapted to calculate and unify the burst detection parameters for the whole network [68]. This method is able to overcome the intrinsic differences between networks leading to a more reliable comparison between the bursting activities.

From the timestamps, we analyzed the SRs (spikes per minute), BRs (bursts per minute), percentages of spikes contained in bursts (spikes in bursts/total spikes), and inter-burst intervals (IBIs) (time in milliseconds from the end of a burst to the start of the subsequent burst). All the above parameters were evaluated for each channel of a MEA and then averaged over the chip. The reported median in the box plots, instead, should be considered as a ratio.

To better visualize the effects of the used drugs on the network activities, we also calculated the fold change of the SRs and BRs. The values before and after stimulation were compared separately for each MEA by dividing the value after stimulation with the respective value at the baseline. We also evaluated the fold change for the baseline conditions by comparing each MEA-specific value to their respective average.

#### 4.7.3. Network Synchronicity by Channel Inter-Correlation Analysis

To evaluate the synchronicity in a network, we used a MATLAB tool called CorSE that analyses the correlations of time-varying spectral entropies between MEA channels [70]. From the tool, we extracted the pairwise inter-channel correlations and connections. Since the correlation is calculated for the channel spectral entropies at lag zero, a high correlation weight corresponds to high synchronization between the networks in the vicinities of the microelectrodes considered. We then analyzed the differences in the weights of the connections between the measurements from the chemically stimulated cultures and their respective baseline measurements for each channel pair. For this specific case, all the correlation weights of each channel pair of each MEA are reported in the results and were not averaged for each MEA.

#### 4.7.4. Network Synchronicity by Binned Activity Analysis

In addition to CorSE, network synchronicity was also assessed based on the spiking activity. We first binned the detected spikes (1 = when a spike was present, 0 = otherwise) that were non-overlapping into 5 ms bins for each MEA channel. After that, we summed the bin values across all MEA channels per bin. Then, we counted all the local maxima of the summed time series. The higher peaks in our summed time series corresponded with spikes and bursts occurring in the same time bin and spanning a larger network. In order to be sure to count the bursts from the summed signal only once, we counted only the peaks separated by at least the minimum IBI found in the MEA from the previous burst analysis. This passage assures us not to count the summed peaks in the same bursts repeatedly. After that, we counted all the remaining detected peaks that spanned more than ten channels, and we defined these as network bursts (NBs).

#### 4.7.5. Delay in the Response Times of the Convulsants

To evaluate the different delays in response times from the 4-AP and gabazine application to detectable responses, we developed an algorithm that is explained below to detect the first burst before the detectable change in bursting pattern. The response of the network to the gabazine exposure produced a visible, highly precise bursting series spanning across the network. Moreover, we visually noticed that the last NBs spanned over half of the electrodes in our cultures and that at the end of the recordings, all our cultures chemically stimulated with gabazine changed their bursting patterns. To assess the differences in the bursting activity before and after detectable drug responses (convulsant-induced bursting), we calculated the mean IBI (*meanIBI*) and the standard deviation of the IBIs (*stdIBI*) of the last 20 NBs in the recording. The choice of the last 20 NBs was made to be sure that the drug would have had time to produce effects on the activity. If there were less than 20 NBs in the recording, the MEAs were discarded for the present analysis. We obtained the normal distribution of the IBIs based on the *meanIBI* and the *stdIBI*. Then, we analyzed the IBIs between the sequential NBs spanning over more than 20 channels. To find the bursts in the repetitive burst series, we used the following rule:

(1) $$\left\{\begin{array}{c} \left|\left.IBI_{i}\left(j\right)-meanIBI_{i}\right|<stdIBI_{i},\;\;\;\;\;NB_{i}\left(j\right)\in repetitive\;series\right.\\ \left|\left.IBI_{i}\left(j\right)-meanIBI_{i}\right|>stdIBI_{i},\;\;\;\;\;NB_{i}\left(j\right)\notin repetitive\;series\right. \end{array}\right.$$

where ***i*** is MEA index, ***j*** is the NB index, and ***stdIBI_i_*** is the standard deviation of the last 20 IBIs of the MEA recording. If the absolute value of the differences between the IBI of an NB and the mean IBI of the corresponding MEA was smaller than ***stdIBI**_i_*, then the NB belonged to the repetitive burst series; otherwise, it did not belong to it. A graphical representation of the selection rule used is shown in Figure 5C.

To finally evaluate the duration it took for the convulsant to show its effects on the neuronal activity, we analyzed the time difference from the convulsant application time point to the time point at which the first burst of the repetitive series occurred and called the time difference a delay.

#### 4.7.6. Statistical Analysis

The raw MEA data were analyzed using MATLAB (v. 2020b), whereas the timestamps and the results were analyzed with SPSS Statistics software (IBM SPSS Statistics for Windows, Version 26.0. Armonk, NY: IBM Corp.), GraphPad Prism software (v. 9) (GraphPad Software, San Diego CA, USA), and MATLAB (v. 2020b). Due to the non-normal distribution of the data, the Mann–Whitney U test or the Wilcoxon rank sum test was used to evaluate differences between culture groups. A *p*-Value under 0.05 was considered significant. Since statistical tests are often affected by large datasets, and thus the *p*-Value quickly tended towards zero even for small differences in the distributions [71], we complemented the significance tests with an effect size analysis [72]. As an effect size measure, we used the Glass’s delta [73] for the comparison between two groups.

In our analysis, the correlation weights at baseline have been used as control. The Glass’s delta value should be interpreted as how many standard deviations the mean value of the chemically stimulated weights is larger than the control mean value; values above 0.8 are normally considered as a large effect size, between 0.2 and 0.8 as a medium effect size, and smaller than 0.2 as a small effect size [74]. The sign of delta indicates which of the two means is higher: a positive value indicates that the mean of the chemically stimulated group is higher than the control, and a negative Glass’s delta means that the mean of the control group is higher. To compute the Glass’s delta, we used the MATLAB toolbox that was made available [75].

## 5. Conclusions

In this study, we aimed to investigate how astrocytes may play a role in the regulation of neuronal activity during drug-induced epileptiform behavior. To do so, we compared the responses of neuron–astrocyte co-cultures with predetermined numbers of neurons and different numbers of astrocytes. We chemically stimulated the co-cultures with 4-AP or gabazine to assess the control of the astrocytes on neuronal behavior. The cultures were plated on MEAs to record the extracellular neuronal electrical activity. This is the first study investigating the effects with respect to different neuron–astrocyte ratios in co-cultures on MEAs and the first study to characterize the effects of 4-AP and gabazine on neuronal networks.

Our results showed that astrocytes effectively counteracted 4-AP-induced epileptic-like activity; in fact, no visible changes in the neuronal activity were noticeable after blocking the voltage-gated K^+^ channels by applying 4-AP. This effect was more detectable when we increased the number of astrocytes, which led to a lower spike rate than at baseline.

On the other hand, the astrocytes did not fully counteract the effect of gabazine, a GABA_A_ receptor (GABA_A_R) blocker. The neuronal activity increased and became synchronized due to gabazine in all cultures. However, this gabazine-induced synchronization was induced with a delay that noticeably extended with the increase of the number of astrocytes in the co-cultures. Thus, the effects of the GABA_A_R blockade were directly connected to the number of astrocytes in the culture, the relative number of GABA_A_Rs, and the extent of the astrocytic network.

Our results further confirm that the astrocyte number in neuronal cultures affects neuronal network maturation and thus functions by enhancing the early development of the neuronal electrical activity. This, together with the astrocyte control of homeostasis, alters the responsiveness of the neurons to the pharmacological agents.

Our study highlights the significant regulatory role of astrocytes on neuron functionality in the brain and the importance of an in detail consideration of the ratio of astrocytes in neuronal cultures exposed to chemical stimulation. Brain regions exhibit inhomogeneous neuron–astrocyte ratios, and this different cellular distribution may reflect differences in epileptic seizure susceptibility. Hence, it is crucial to explore the impact of the drugs in co-cultures with different cellular ratios, as was done in this paper.

## Figures and Tables

**Table 1:** Summary table of the methods and results. (NS: a cell culture without explicitly added astrocytes; 90/10, 80/20, 70/30, 50/50: percentages of plated neurons/astrocytes for each cell culture type; NB: network-wide burst; ICC: immunocytochemistry).

Cell Culture Platform	Cell Culture Type	n	Experiment/Analysis	Methods Section	Stimulation (4-AP 75 µM or Gabazine 30 µM)	Results Section	Results
**MEAs**	NS, 90/10, 80/20, 70/30, and 50/50	8 MEAs per cell culture type (total n = 40)	Spontaneous baseline activity recording	4.6	None (control)	2.2 and 2.3	NS cultures experienced more robust development at the later stages of culture (DIV21-28) compared to co-cultures. Development of electrical activity was more robust for co-cultures with higher ratios of astrocytes (70/30 and 50/50) at early stages of culture (DIV7-DIV14). Co-cultures with higher astrocyte numbers had lower spike and burst rates and a lower percentage of spikes in bursts. IBI increased with increasing neuron–astrocyte ratio.
			Acute stimulation recording	4.7	4-AP	2.3	Slight increase in SRs and BRs for NS, 90/10, and 80/20, whereas with 70/30 and 50/50 SRs remained mostly unchanged, and the BRs slightly decreased. The percentage of spikes in bursts was reduced in all the cultures, and the IBI and its variability were slightly increased.
					Gabazine	2.3	SRs and BRs increased in all cultures. IBI values decreased for all cultures, and the percentage of spikes in bursts increased, synchronizing the network activity.
			Washout followed by recording after 24 h	4.7	4-AP	2.3	SRs were generally lower than at baseline except for NS and 70/30. BRs were higher than at baseline for NS and all the co-cultures except 80/20.
					Gabazine	2.3	SRs and BRs were generally lower for all cultures, except in the case of BRs in 90/10.
			Cross-correlation	4.7.3	4-AP	2.4	Either no change or a small decrease in the numbers of channels with synchronized activity and the correlation weights of the connections.
					Gabazine	2.4	Increased connection weights in all co-cultures which shifted the weight difference distribution towards positive values.
			NBs	4.7.4	4-AP	2.4 and 2.5	The number of network-wide bursts decreased.
					Gabazine	2.4 and 2.5	Repetitive synchronous NBs spanned over a constant number of channels (~40 channels).
			Convulsant response delay	4.7.5	4-AP	2.5	No noticeable delays in the NB synchronization.
					Gabazine	2.5	Increasing the relative numbers of astrocytes drastically delayed the effects of gabazine on neuronal firing (approximate delays were NS: 25 s; 90/10: 130 s; 80/20: 150 s; 70/30: 180 s; 50/50: 230 s).
**Coverslips**	NS, 90/10, 80/20, 70/30, and 50/50	3–4 per staining	ICC	4.3	None (control)	2.1	Neuron–astrocyte ratios were coherent after DIV14 with plating densities, and neurons counts were homogeneous through all the coverslips counted.
**Coverslips**	NS	3–4	ICC	4.3	Control/4-AP/Gabazine	2.3	4-AP slightly increased the neuronal viability. Neither 4-AP nor gabazine were toxic to neurons and did not decrease cell viability or affect their appearance.
		3–4	Live/dead assay	4.4			
**24-Well Plates**	Astrocytes	2–3	ICC	4.3	Control/4-AP/Gabazine	2.3	No differences in cell viability or appearance.
		2–3	Live/dead assay	4.4			

## Data Availability

The code for the burst analysis is available at: The code for the correlated spectral entropy analysis (CorSE) is available in Matlab Central at: https://www.mathworks.com/matlabcentral/fileexchange/59626-spectral-entropy-based-neuronal-network-synchronization-analysis-corse, (accessed on 24 November 2021).

## Supplementary Materials

The following are available online at https://www.mdpi.com/article/10.3390/ijms222312770/s1.
