# Supplementary material for: Astrocytes Exhibit a Protective Role in Neuronal Firing Patterns under Chemically Induced Seizures in Neuron–Astrocyte Co-Cultures
Source: Int J Mol Sci. 2021 Nov 25;22(23):12770. doi: 10.3390/ijms222312770 (PMC8657549; doi:10.3390/ijms222312770)
Supplement: Supplementary file 1 [file ijms-22-12770-s001.zip › ijms-1463214-suppl.pdf]

International Journal of
Molecular Sciences

MDPI

Supplementary data

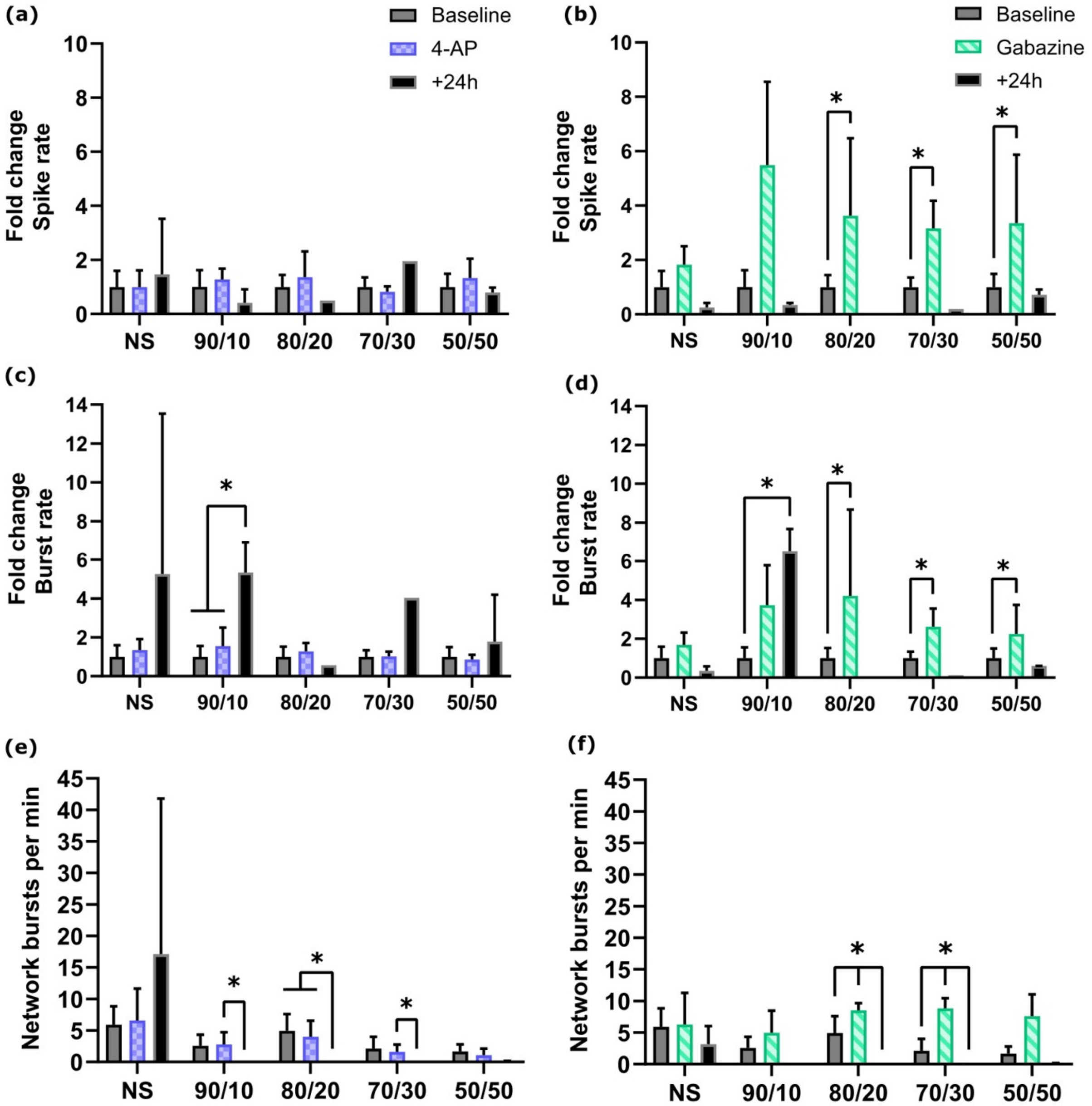

Figure S1. Fold changes of the SR and BR at baseline at DIV28 (in gray), immediately after the chemical stimulation (4-AP in lilac, gabazine in green) and after 24 hours (in black), along with the network burst rates, for the (**a**) (**c**) (**e**) 4-AP and (**b**) (**d**) (**f**) gabazine stimulations. – * $p < 0.05$.

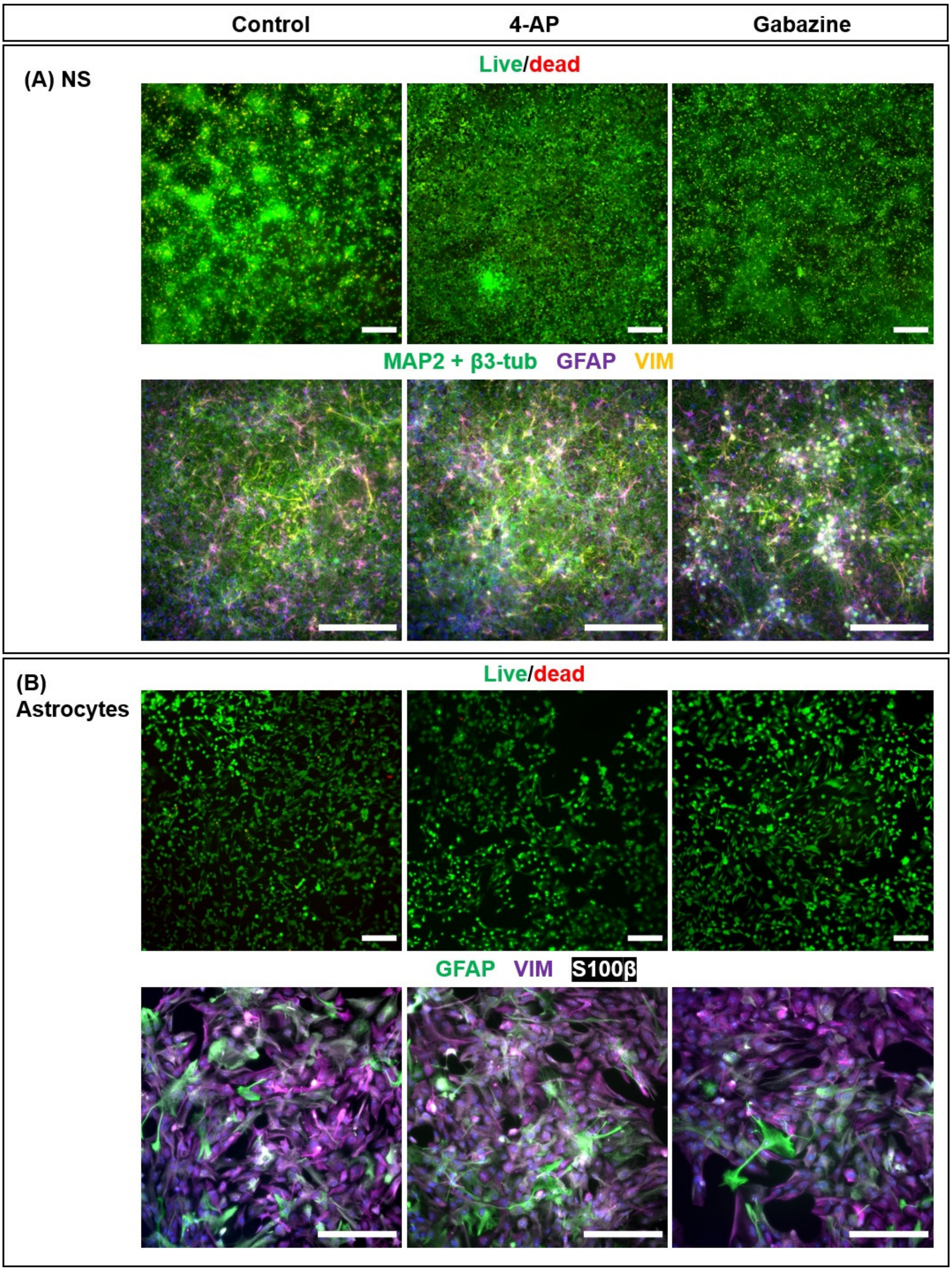

Figure S2. Live/Dead and ICC assay of NS (DIV14) **(a)** and astrocyte (DIV4) **(b)** cultures after 24-hour 4-AP or gabazine stimulation. (**a**) 4-AP slightly increased neuronal viability compared to untreated control cultures, whereas gabazine did not affect viability. Pharmacological stimulation did not affect neuronal appearance or expression of neuronal (MAP2 + β3-tubulin) proteins in the culture. Cultures also included small amounts of spontaneous astrocytes (GFAP and VIM). (**b**) There were no changes in astrocyte viability after 24-hour stimulation with 4-AP or gabazine. Furthermore, astrocytes maintained their typical morphology and expression of astrocytic proteins (GFAP, S100β, and VIM) after stimulation. – Scale bar 200 μm in all images.

Table S1. Effect size analysis of 4-AP and gabazine for NS and co-cultures. The effect of 4-AP and gabazine depended on the number of astrocytes in the culture. The second and third columns show the Glass's delta for the difference between the correlation weights distribution after stimulation and the correlation weight distribution at baseline, respectively for 4-AP and gabazine. Values above 0.8 were considered large effect size, between 0.2 and 0.8 medium effect size, and smaller than 0.2 small effect size. Large effect sizes are indicated in bold. The fourth and fifth columns represent the confidence intervals of the Glass's delta.

	**Glass's Delta**		**Confidence Intervals**	
**Cell Culture**	**4-AP**	**Gabazine**	**4-AP**	**Gabazine**
**NS**	0.14	0.15	[0.1; 0.18]	[0.11; 0.19]
**90/10**	0.20	0.69	[0.17; 0.24]	[0.64; 0.74]
**80/20**	-0.03	**1.30**	[-0.06; 0.004]	[1.26; 1.34]
**70/30**	0.58	**1.14**	[0.54; 0.61]	[1.10; 1.18]
**50/50**	0.05	**0.94**	[0.008; 0.08]	[0.89; 0.98]
